# Mutual Support of Ligand- and Structure-Based Approaches—To What Extent We Can Optimize the Power of Predictive Model? Case Study of Opioid Receptors

**DOI:** 10.3390/molecules26061607

**Published:** 2021-03-14

**Authors:** Sabina Podlewska, Rafał Kurczab

**Affiliations:** 1 Department of Technology and Biotechnology of Drugs, Jagiellonian University, Medical College, 9 Medyczna Street, 30-688 Cracow, Poland; smusz@if-pan.krakow.pl; 2 Maj Institute of Pharmacology, Polish Academy of Sciences, 12 Smętna Street, 31-343 Cracow, Poland

**Keywords:** machine learning, docking, opioid receptors, in silico drug design and discovery

## Abstract

The process of modern drug design would not exist in the current form without computational methods. They are part of every stage of the drug design pipeline, supporting the search and optimization of new bioactive substances. Nevertheless, despite the great help that is offered by in silico strategies, the power of computational methods strongly depends on the input data supplied at the stage of the predictive model construction. The studies on the efficiency of the computational protocols most often focus on global efficiency. They use general parameters that refer to the whole dataset, such as accuracy, precision, mean squared error, etc. In the study, we examined machine learning predictions obtained for opioid receptors (mu, kappa, delta) and focused on cases for which the predictions were the most accurate and the least accurate. Moreover, by using docking, we tried to explain prediction errors. We attempted to develop a rule of thumb, which can help in the prediction of compound activity towards opioid receptors via docking, especially those that have been incorrectly predicted by machine learning. We found out that although the combination of ligand- and structure-based path can be beneficial for the prediction accuracy, there still remain cases that cannot be reliably predicted by any available modeling method. In addition to challenging ligand- and structure-based predictions, we also examined the role of the application of machine-learning methods in comparison to simple statistical methods for both standard ligand-based representations (molecular fingerprints) and interaction fingerprints. All approaches were confronted in both classification (where compounds were assigned to the group of active and inactive group constructed on the basis of K_i_ values) and regression (where exact K_i_ value was predicted) experiments.

## 1. Introduction

Computational methods are now an indispensable element of the drug design process, being used at all stages–from ligand identification via its optimization (both in terms of activity and properties) to monitoring its effect after introduction into the market [1,2]. A wide range of approaches applied to find new potential drug candidates can be divided into those that use only information about existing ligands (ligand-based methods [3,4,5,6,7,8]) and those that take into account the structure of the target protein (structure-based methods [9,10,11,12,13,14]). The former group of approaches has the following representatives: similarity searching, pharmacophore modeling, quantitative structure–activity relationship analysis (QSAR), etc., whereas structure-based drug design focuses on docking to the three-dimensional structure of the target protein. In the optimistic case, it is available from the crystal structure; however, for the great majority of target proteins, such data are unavailable. In such a situation, the atom arrangement of the target needs to be predicted, which is most often achieved via the homology modeling procedure [15].

Both ligand- and the structure-based path have their advantages and disadvantages. Ligand-based approaches are, in general, faster and are less demanding in terms of computational resources. However, as the predictions are based on models built on the known ligands, the quality of the obtained results depends on the quality of the training data available. For example, when the set of known ligands is small, and it is composed of compounds sharing high structural similarity, the predictive model may have difficulties in the correct evaluation of highly diversified compound libraries [16,17]. On the other hand, the structure-based methods are less prone to bias related to the training set, although they are much more demanding in terms of computational resources. In addition, the discrimination ability between active and inactive compounds also varies for different targets, depending, e.g., on the size and properties of the binding site.

The most common ligand-based strategies involve similarity searches, pharmacophore modeling, and QSAR analysis. Both similarity searches and QSAR analysis often make use of machine learning (ML) methods. They are very popular in the computer-aided drug design (CADD) field due to their speed and relatively high-efficiency of compound properties predictions. Nevertheless, the ML-based predictions are prone to bias related to many factors, from the training set composition, via compound representations to results evaluations [17].

The effectiveness of various computational methods depends on the target, already available ligands, and method settings. The most often conducted studies on the efficiency of computational protocol predictions focus on global prediction efficiency. They use general parameters that refer to the whole dataset, such as accuracy, Matthews correlation coefficient (MCC), precision, mean squared error (MSE), etc. [18,19,20,21,22,23,24,25]. Here, we scrupulously examine ML predictions obtained for opioid receptors (mu, kappa, delta). We do not focus on global prediction efficiency but carefully analyze cases for which the predictions were the most accurate and the least accurate, and by using other methods, we try to explain prediction errors.

Opioid receptors are representatives of the G protein-coupled receptors (GPCRs), being the largest and the most diverse proteins in the human genome [26,27,28]. Opioid receptors are responsible mainly for analgesia, and therefore they constitute intensively explored targets for pain treatment [29]. Their three main subtypes (mu-opioid receptor, kappa-opioid receptor, and delta-opioid receptor) are involved in many physiological processes in the living organism. Although the function of opioid receptors in the brain is still not fully explained, they are proved to play an important role in obesity, respiratory and cardiovascular control, epileptic seizures, emotional response, and regulation of membrane ionic homeostasis [30,31,32,33,34].

There is already a great collection of studies comparing the quality of ML-based predictions made in various conditions. However, such comparisons are usually based on the global prediction efficiency [35,36,37]. In the study, we focus on more detailed analysis and carefully examine cases with the highest prediction error. Such an approach was applied to see whether there is room for improvement of the prediction quality using different experimental settings (e.g., compound representation) or evaluation strategy (e.g., shift from ML to docking). Moreover, to provide also a more general picture of the considered problem, ML approaches were confronted with simple statistical methods in both classification and regression tasks.

## 2. Methods

### 2.1. Dataset Preparation

Respective ligand sets were prepared based on the ChEMBL database (version 25, European Molecular Biology Laboratory-European Bioinformatics Institute, Cambridgeshire, UK) [38]. All affinity values (expressed via K_i_) referring to mu, kappa, and delta-opioid receptors were collected. The compound structures were transformed to the bit-string representation using the PaDEL descriptor [39] software (version 2.17, National University of Singapore, Singapore ) (the following fingerprints were used: extended fingerprint (ExtFP) [40], Klekota–Roth fingerprint (KlekFP) [41] and MACCS fingerprint (MACCSFP) [42]).

### 2.2. ML-Based Predictions

K_i_ values were predicted using the k-nearest neighbor algorithm (IBk [43]) and random forest (RF) [44,45]. K_i_ values were predicted in regression (exact K_i_ value was predicted) and classification (assignment to the active, K_i_ < 100 nM, or inactive, K_i_ > 1000 nM, class) experiments. Predictions were carried out in the 10-fold cross-validation mode with random division into folds. Weka’s (version 3.6.10, University of Waikato, Hamilton, New Zealand) implementation of the ML algorithms was used [46].

### 2.3. Molecular Docking

In the second path, all the compounds were docked to the respective crystal structures of opioid receptors (Table 1). The crystal structures were prepared for docking using the Protein Preparation Wizard from the Schrödinger Suite, and the docking was carried out in Glide [47] from the same software package in the extra precision mode. The compound’s three-dimensional conformations were generated within LigPrep [48] with the use of the OPLS3 force-field.

The obtained ligand–receptor complexes were encoded in the form of the structural ligand interaction fingerprints (SIFts) [49]. Those positions for which the ligand-residue contacts occurred for more than 50% of ligands were analyzed in terms of the contact frequency (the groups of active and inactive compounds were analyzed separately). In addition, regression experiments predicting K_i_ values were carried out in an analogous manner as for ExtFP, MACCSFP and KlekFP.

## 3. Results and Discussion

### 3.1. Dataset Analysis

**Figure 1:**
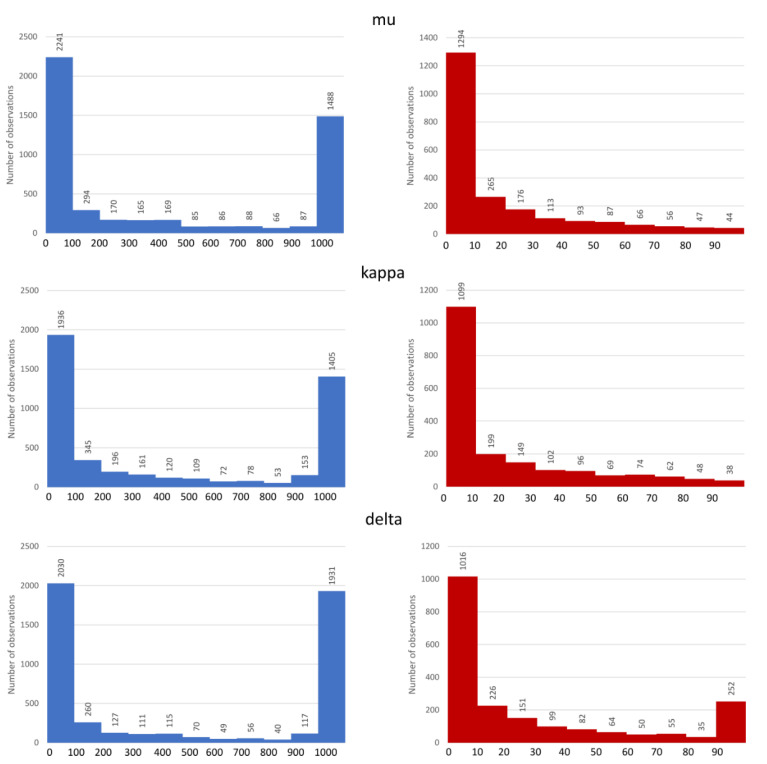
Distribution of K_i_ values of opioid receptors ligands, full data are depicted in blue, red bars refer to the activity distribution of the most active ligands (K_i_ < 100 nM).

The number of examples considered for a particular target is as follows: 4939 datapoints for the mu opioid receptor, 4628 for the kappa subtype, and 4906 for the delta-opioid receptor. The activity distribution for considered targets is presented in Figure 1.

The first observation coming from Figure 1 is the relatively high number of very active ligands (K_i_ < 10 nM) reported in the ChEMBL database. For all receptors considered, nearly half of the data points refer to records with K_i_ values below 100 nM. Moreover, when taking a closer look at them, it appears that also the majority of them can be assigned to the group of very active ligands, that is, below the 10 nM. For all three receptors, there are over 1000 ligands with K_i_ values below the 10 nM threshold.

### 3.2. Global Effectiveness of ML Methods Predictions (Regression Experiments)

#### 3.2.1. Ligand-Based Analysis

**Figure 2:**
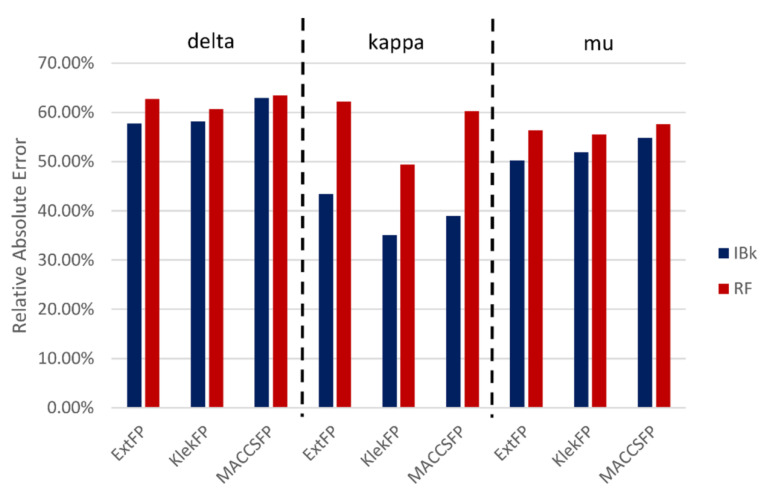
Relative absolute errors were obtained in the predictions of compounds K_i_ values for different machine learning (ML) algorithms and compound representations.

The global effectiveness of such a strategy is presented in Figure 2, and it is expressed in the form of relative absolute error.

The general observation is that the predictions of the RF algorithm were a little bit less accurate than those provided by IBk (indicated by higher values of Relative Absolute Error). Moreover, for the kappa opioid receptor, the differences between results obtained by RF and IBk are the highest and equal to about 20%. Although, on average, the best predictions were obtained for KlekFP, only for the kappa opioid receptor, the difference between various compound representations is not strongly indicated. When it comes to the analysis of a particular receptor subtype, the most accurate results were obtained for the kappa opioid receptor, with values of relative absolute error not exceeding 50% for all compounds representations for IBk, and between 50% to 60% for RF. On the other hand, the lowest prediction power was observed for the delta opioid receptor, where Relative Absolute Error values were around 60% for all ML methods and compound representations used–the highest for MACCSFP, with only a slight difference between IBk and RF.

**Figure 3:**
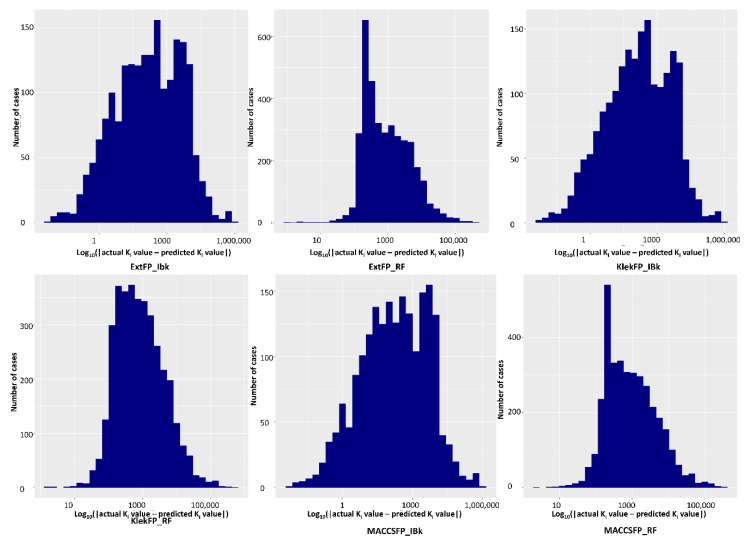
Distribution of prediction error for the delta-opioid receptor.

In addition, for each case, the distribution of prediction error was provided (example distribution for the delta opioid receptor is visualized in Figure 3, respective data for remaining targets are placed in the Supplementary Materials). It is visible that for each method, there is a peak in the prediction error. It is most visible for ExtFP and MACSFP-based RF models, and in both cases, it is between 100 and 1000 nM error with over 600 such cases for ExtFP and 400 for MACCSFP. Predictive models constructed on ExtFP and MACCSFP representations using the IBk algorithm did not have such sharp peaks in predictive error, and its relatively high values are observed for 100 to 10,000 nM. KlekFP representation displayed different behavior; for RF, the highest number of prediction errors was of values around 1000. On the other hand, the range of the highest populated error values was much broader than in the case of MACCSFP and ExtFP. The distribution error for KlekFP–IBk resembled a distribution error for ExtFP–IBk.

In addition, regression experiments were carried out for compounds represented by interaction fingerprints (SIFts). However, in this case, the prediction accuracy was much lower, as relative absolute error values exceeded 90%.

**Figure 4:**
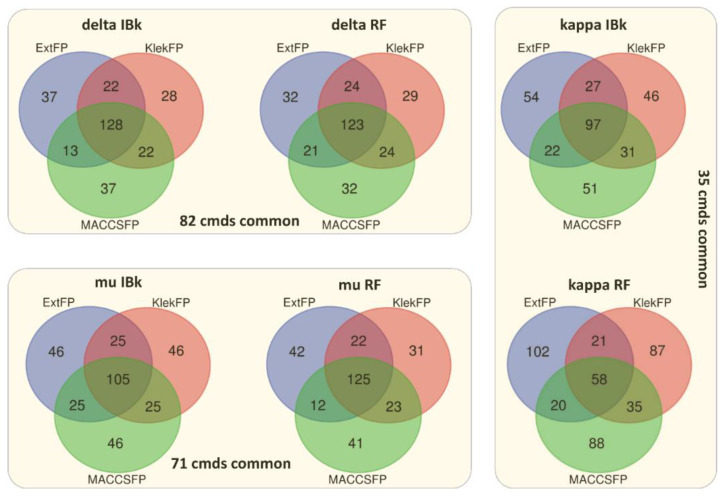
Overlap of the top 200 compounds with the highest prediction error.

To analyze whether the compounds for which the ML methods are unable to produce correct predictions are the same for different methods/compound representations, Venn diagrams presenting the number of overlapping compounds for each experimental setting were prepared (200 top compounds were considered in each case, Figure 4). There is no direct tendency when IBk and RF algorithms are compared. Although for delta and kappa receptors, the highest number of common compounds with the highest error occur for IBk, the highest number of ligands that were consistently incorrectly predicted for mu opioid receptor occurred for RF. The highest number of compounds consistently incorrectly predicted for both representations and ML methods occurred for the delta opioid receptor (82 compounds). It was similar to the number of wrongly predicted ligands from the set of mu opioid receptors (71), whereas for the kappa opioid receptor, the number of ligands, which were incorrectly predicted in all experimental conditions, was much lower, and it was equal to 35. Therefore, for delta and mu receptors, the relatively high percentage of compounds (over 35%) is wrongly predicted regardless of the compound representation and ML method used. Therefore, it can be assumed that for these compounds, the ligand-based approach is ineffective in the correct evaluation of their activity. For kappa opioid receptors, almost 50% of compounds (out of the top 200 worst predictions) were wrongly predicted by IBk for all fingerprints used. In contrast, RF managed to lower this number to 17%, which means that in the case of these receptor ligands, an improvement in prediction efficiency can be obtained by the use of other ML algorithms.

To characterize the set of wrongly predicted compounds in more detail, several analyses were carried out. At first, it was checked whether the compounds for which the highest predictive error was consistently obtained belong to the group of active or inactive ligands (Table 2). Except for the mu opioid receptor, where over 50% of incorrectly predicted compounds belonged to the group of active ligands, the distribution of compounds over two activity groups considered (active/inactive) was similar. It was varying from 30–40% (the obtained numbers do not sum to 100%, as there is a gap in K_i_ values when dividing the dataset into the active (K_i_ below 100 nM) and inactive (K_i_ above 1000 nM) parts. This outcome is promising in terms of the potential application of the tested methods in VS, as the most common problem occurring in compound evaluation is results bias. It is most often related to compound structures (those that are present in the training set do not resemble representatives from the test set), but it can also be related to the overrepresentation of one of the acting classes in the training set. Then, there is a higher probability that the newly evaluated examples will be assigned to the class with the highest number of examples regardless of compound structure (although this problem can be solved via the application of a proper weighting scheme).

**Figure 5:**
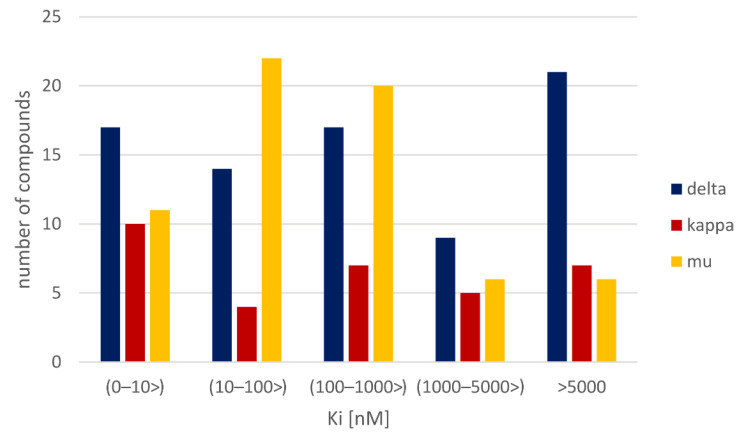
K_i_ distribution for compounds with the highest prediction error common for all methods used.

As the second type of analysis, a more detailed examination of the activity distribution of incorrectly predicted compounds was carried out (Figure 5). In general, for mu-opioid receptor ligands, the highest populated (in terms of consistently incorrectly predicted compounds) activity range was between 10 and 100 nM and 100 to 1000 nM (when activity was expressed in the form of K_i_ values). On the other hand, compounds with relatively high K_i_ (over 1000 nM) constituted only a small fraction of mu-opioid receptor ligands with the highest prediction error. On the other hand, delta-opioid receptor ligands were characterized by the highest prediction error when the compound K_i_ values were above 5000 nM. For kappa opioid receptor ligands, the results were also different as in the case of this receptor, the highest number of compounds with the highest prediction error fell to the range of K_i_ below 10 nM. Due to high variations in the percentage of incorrectly predicted compounds falling to a particular range of K_i_ values, no general conclusions can be drawn in terms of compound activity ranges, for which the highest difficulties in proper evaluation by ML methods occur.

#### 3.2.2. Structure-Based Analysis

**Figure 6:**
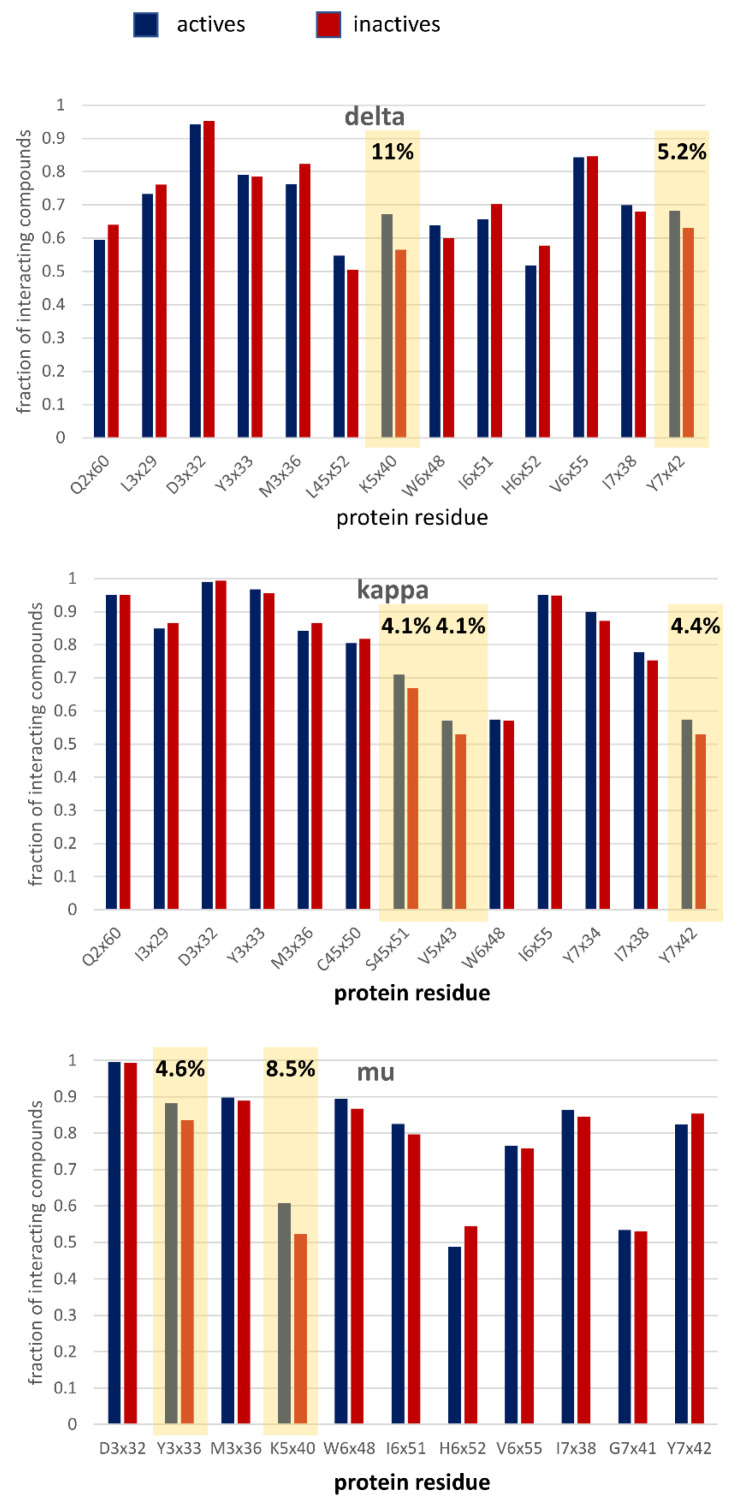
Histograms of the frequencies of interactions of compounds with particular amino acids (only positions with interaction frequency above 50% are shown, the formation of any contact is considered). Positions with the highest difference between active and inactive compounds are indicated.

To explain the observed dependencies from the structure-based point of view, the docking studies for all the analyzed compounds were carried out. The obtained ligand–receptor complexes were encoded in the form of the SIFts [49], and those positions, for which the ligand-residue contacts occurred for more than 50% of ligands were analyzed in terms of the contact frequency (the groups of active and inactive compounds were analyzed separately). The analysis allowed for the identification of positions with the highest difference between the interaction frequency of active and inactive compounds (Figure 6).

For the delta opioid receptor, the K5x40 residue interacted by 11% more frequently with active ligands than the inactive ones. In addition, Y7x42 displayed a preference for active compounds, but the difference in the interaction frequency was much lower and equal to 5.2%. Moreover, all the positions with the highest difference in interaction frequencies between the analyzed compound groups for kappa and mu opioid receptors displayed a preference for active compounds. For the kappa opioid receptor, there were three residues with a difference at the level of 4% (S45x51, V5x43, and Y7x42) and two for the mu-opioid receptor (Y3x33 and K5x40). Although the residues discriminating active and inactive compounds belong to different protein regions, it seems that the 5th transmembrane helix is the most discriminative in this case.

**Figure 7:**
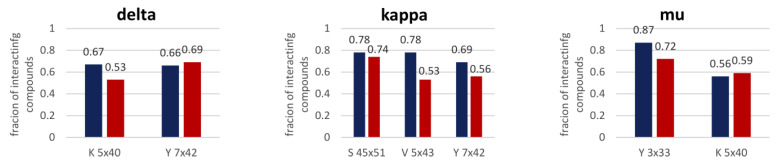
Histograms of the frequencies of interactions of compounds with selected amino acids for compounds with the highest prediction error.

The compounds with the highest prediction error were compared (Figure 7) in terms of the interaction frequency with amino acids detected in Figure 6.

The analysis performed in Figure 7 indicates that for some fraction of the dataset, active compounds interact more frequently with amino acids selected in Figure 6; therefore, these contacts can be used for the correct determination of compound activity. Nevertheless, for most of them, contact patterns between actives and inactives do not allow for correct assignments to the activity class. Consequently, via this approach, only several percentages of incorrectly predicted compounds can be correctly re-evaluated.

### 3.3. Classification Experiments

**Figure 8:**
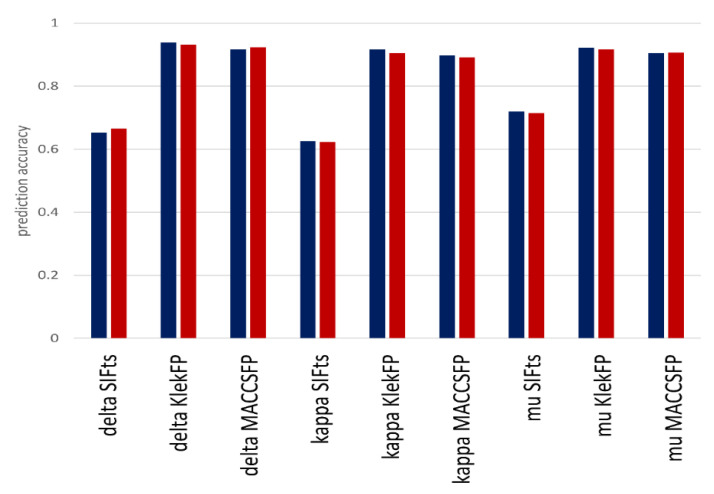
Prediction accuracy obtained for ML models constructed on key-based fingerprints and interaction fingerprints. Dark blue: k-nearest neighbor algorithm (IBk), red: random forest (RF).

In order to make a comprehensive comparison of methods used for compound evaluation, the ML models were also constructed on the IFP data, and classification experiments were carried out. Classification experiments are directly related to the problem presented in Figure 6, as the construction of ML models is mostly based on the differences in the feature values for different groups (in order to make it possible to make a distinguishment between them). The efficiency of ML-based division into active and inactive (expressed as prediction accuracy) compounds is presented in Figure 8.

(1) $$prediction\;accuracy=\;\frac{number\;of\;correct\;predictions}{number\;of\;all\;predictions}$$

The most important observation coming from Figure 8 is that the power of ML methods performance was much higher when standard ligand-based fingerprints were used for compound representation rather than when they were encoded in the form of interaction fingerprints. The difference is over 25% in terms of accuracy. Moreover, although it was a delta opioid receptor, for which the highest differences in the contact frequency for active and inactive compounds occurred, the ML methods were the most effective in the proper compound assignment to particular activity class for the mu opioid receptor (it was the only target for which the prediction accuracy on IFP exceeded 70%). When it comes to more general observations, predictions for KlekFP were more effective than those for MACCSFP, and IBk was, in most cases, more effective than RF.

Although predictions of the particular value of the K_i_ parameter were sometimes related to high prediction error, considering the problem more generally and focusing on dividing compounds into two activity classes seem to be a more effective strategy than making attempts to predict the exact K_i_ value of a compound.

As already mentioned, additional regression experiments carried out for interaction fingerprints confirmed hits observation, as relative absolute error values for regression on SIFts exceeded 90% (whereas for ExtFP, MACCSFP and KlekFP, they ranged from ~30–60%).

**Figure 9:**
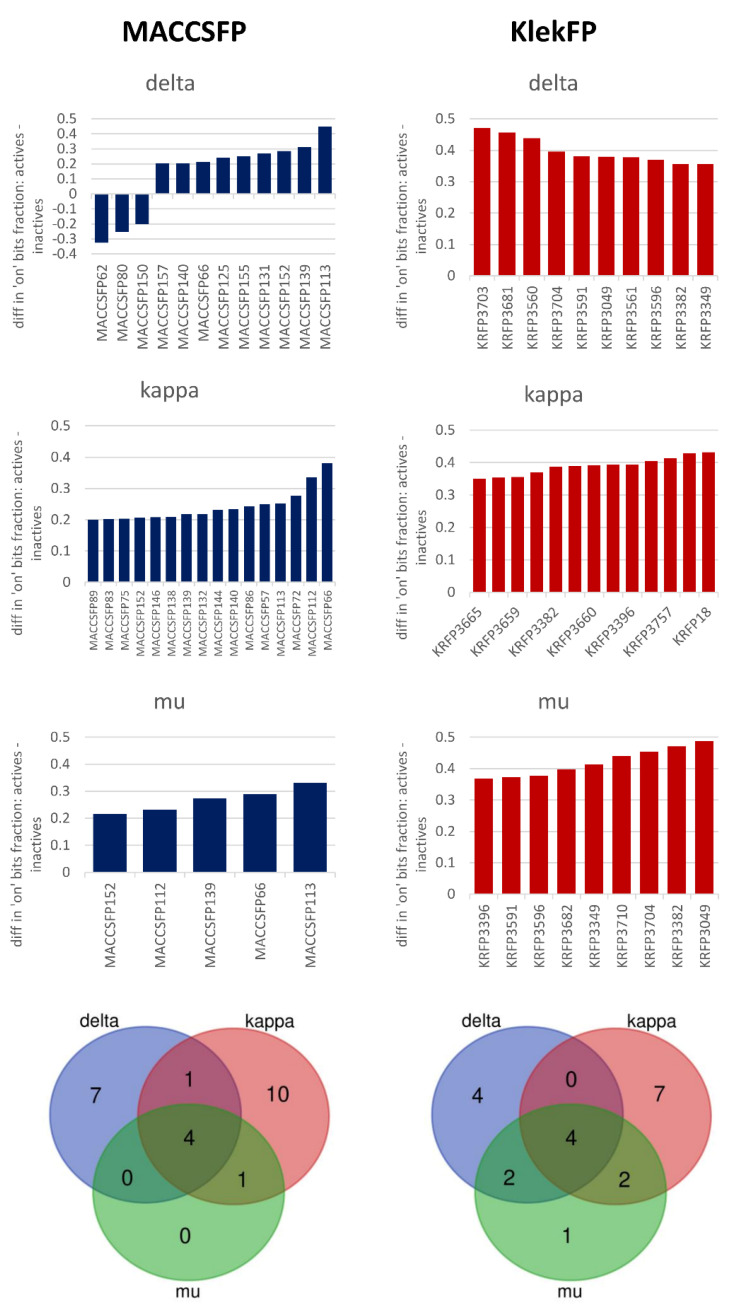
Keys with the highest differences in “on” bit frequency between active and inactive compounds. Venn diagram presents overlap of keys for different targets considered.

In addition to classification experiments on ligand-based data, simple statistics on key-based fingerprints were carried out. Their outcome is presented in Figure 9. The figure presents keys with the highest differences in “on” bit occurrences between active and inactive compounds–for MACCSFP, the threshold was set to 20%, for KlekFP–to 35%. The first observation coming from the figure is that the number of keys providing the highest differentiation between active and inactive compounds varies for different targets. For both MACCSFP and KlekFP, the highest number of keys above the threshold occur for the kappa-opioid receptor and the lowest for the mu opioid receptor. In the latter case, for MACCSFP, the number of keys meeting the criterium of a minimum of 20% of the difference between active and inactive compounds in terms of “on” bits frequency is equal only to 5. Interestingly, out of these five keys indicated for receptor mu, four of them were also highly discriminative for kappa and delta, which may provide general rules for activity within the family of opioid receptors.

Another important observation is that for the standard fingerprints, the differences between “on” bits frequencies for active and inactive compounds are much higher than for SIFts (there is also a tendency is that they are higher for KlekFP than for MACCSFP). For each target considered, there are several keys for KlekFP representation, which enable discrimination of over 40% of compounds, which is a significantly higher number than several percentage differences observed for SIFts. The outcome of simple statistics is also reflected by the performance of ML models, which were much more effective in active/inactive compound classification for key-based fingerprints than for interaction fingerprints.

**Figure 10:**
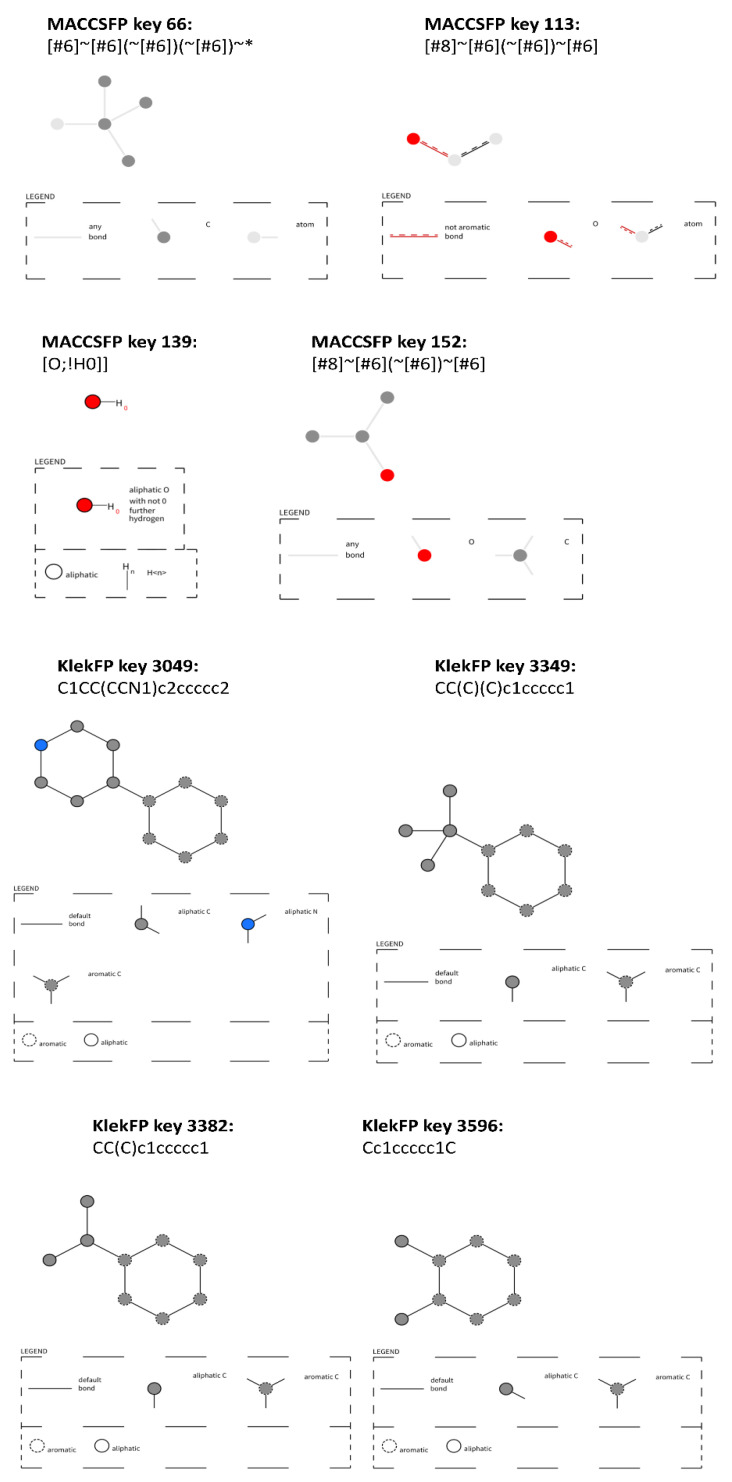
Keys with the highest difference in “on” bits common for all opioid targets considered. Visualized with the use of https://smarts.plus/ SMART.plus.co (accessed on 25 February 2021).

Keys with the highest discriminative potency, which were common for all three opioid targets, are visualized in Figure 10.

The obtained results show a significant difference between the keys indicated by MACCSFP and KlekFP. For MACCSFP, they are more general; in the majority of cases, they include oxygen, and they do not contain aromatic moieties. On the other hand, all indicated keys from KlekFP contain aromatic moieties, mono- or disubstituted by other substructures composed of aliphatic carbons. Due to the fact that the keys indicated for KlekFP are less general, they enable better discrimination between active and inactive ligands of opioid receptors.

### 3.4. Case Studies

**Figure 11:**
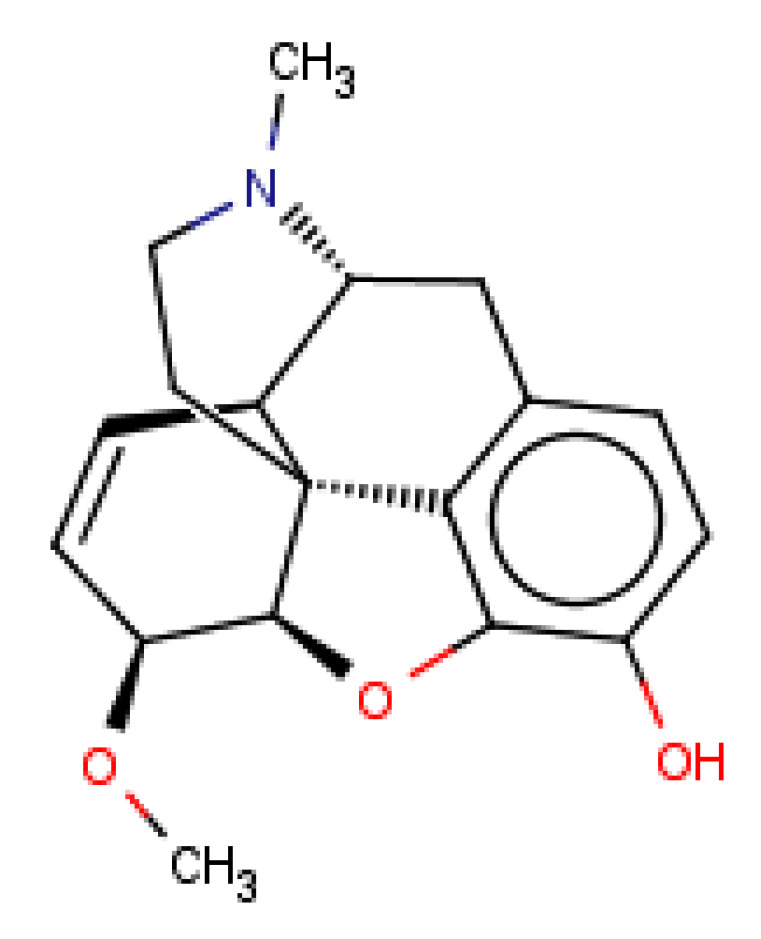
Chemical structure of delta-opioid receptor–ligand CHEMBL358043 with K_i_ equal to 65.80 nM (docking score: −4.12).

Finally, a detailed analysis of particular case studies was carried out. We would like to see for particular compounds whether the structure-based path can help in the improvement of the correctness of the activity prediction by ML methods. The compound taken for the case study was delta opioid receptor–ligand–CHEMBL358043 (Figure 11) with K_i_ equal to 65.80 nM, whereas the predicted K_i_ was over 10,000 nM higher.

At first, the set of structurally similar compounds within the set of considered ligands was identified (Table 3).

**Figure 12:**
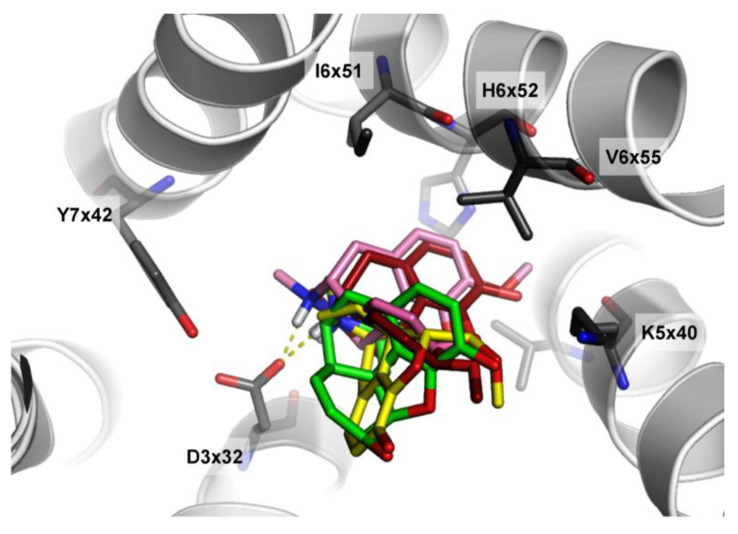
Docking results of CHEMBL358043 (yellow), CHEMBL409938 (green), CHEMBL369475 (pink), and CHEMBL3923831 (firebrick) in the binding site of the delta-opioid receptor (PDB ID: 4RWD). The yellow dotted lines represent the hydrogen bonds.

CHEMBL358043, as well as its analogs presented in Table 3, were examined in terms of their fitting in the binding site of the delta opioid receptor. It is visible that the automatic evaluation via the docking score values also does not provide an efficient estimation of compound activities. The analysis of docking results of selected compounds is presented in Figure 12.

The docking results show that the shift to the structure-based approach would not help in the prediction efficiency of CHEMBL358043, as its docking pose is most similar to orientations of inactive compounds, rather than the most active CHEMBL2409938.

## 4. Conclusions

The great applicability of ML methods in computer-aided drug design tasks forces careful analysis of its predictive power before a method can be used in real applications. In the study, we considered the problem of the ML method’s power in the prediction of a compound’s potential bioactivity not generally but rather more locally and tried to analyze cases with the most difficulties of obtaining correct predictions. The correctness of compound activity prediction was more related to the ML method algorithm than compound representation, as there was a much higher overlap of compounds with high prediction error for different representations than for different ML algorithms. The correctness of activity prediction was also more related to compound structure than its activity (the distribution of prediction error was similar for different activity ranges). Although the use of structural data intuitively should help in achieving better results, our study indicates that it can be obtained only for some fraction of compounds. There still remains a high number of compounds for which even the application of docking does not provide sufficient information for their correct evaluation. Moreover, the value of docking studies seems to be higher when their outcome is not evaluated automatically, e.g., with the use of interaction fingerprints, but manually, using chemical knowledge and information about desired ligand–protein contacts. Therefore, ligand-based approaches are a good starting point for virtual screening campaigns due to their speed in comparison to structure-based methods, such as docking. In addition, although in ML-based applications, standard molecular fingerprints were more effective than interaction fingerprints, the docking studies undeniably consist of a resource of incredibly valuable knowledge on possible compound activity.

## Figures and Tables

**Table 1:** The summary of the crystal structures of opioid receptors used in the study.

Target	PDB ID	Resolution (Å)	Co-Crystallized Ligand Type	Receptor State
Mu opioid receptor	4DKL	2.8	Antagonist	Inactive
Delta opioid receptor	4RWD	2.7	Antagonist	Inactive
Kappa opioid receptor	6B73	3.1	Agonist	Active

**Table 2:** Comparison of the common compounds with the highest prediction error belonging to the group of active and inactive compounds.

Target	Total Number of Common Compounds with the Highest Error	Number of Common Compounds Belonging to the Set of Active Molecules (Fraction of All Common)	Number of Common Compounds Belonging to the Set of Inactive Molecules (Fraction of All Common)
Delta opioid receptor	82	31 (38%)	31 (38%)
Kappa opioid receptor	35	14 (40%)	12 (34%)
Mu opioid receptor	71	37 (52%)	13 (18%)

**Table 3:** The set of structurally related compounds to CHEMBL358043 present in the delta-opioid receptor dataset.

ChEMBL ID	Chemical Structure	K_i_ (nM)	Docking Score	Tanimoto Coefficient towards ChEMBL358043
CHEMBL3923831	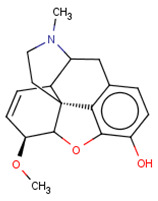	4297	−5.23	1.0
CHEMBL412301	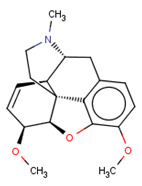	4410	−3.93	0.996
CHEMBL409938	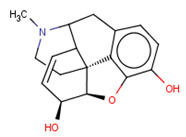	11	−4.14	0.988
CHEMBL369475	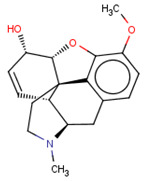	5000	−5.16	0.984
CHEMBL485	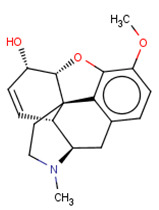	28,438	−4.13	0.984

## Data Availability

The data used in this study are openly available in the ChEMBL database (https://www.ebi.ac.uk/chembl/).

## Supplementary Materials

The following are available online.

## Abbreviations

QSAR	Quantitative structure–activity relationship
ML	Machine learning
CADD	Computer-aided drug design
MCC	Matthews correlation coefficient
MSE	Mean squared error
GPCRs	G-protein-coupled receptors
ExtFP	Extended fingerprint
KlekFP	Klekota–Roth fingerprint
MACCSFP	MACCS fingerprint
IBk	k-nearest neighbor algorithm
RF	Random forest
SIFts	Structural interaction fingerprint
