# Supplementary figures and images for: Mutual Support of Ligand- and Structure-Based Approaches—To What Extent We Can Optimize the Power of Predictive Model? Case Study of Opioid Receptors
Source: Molecules. 2021 Mar 14;26(6):1607. doi: 10.3390/molecules26061607 (PMC7998793; doi:10.3390/molecules26061607)

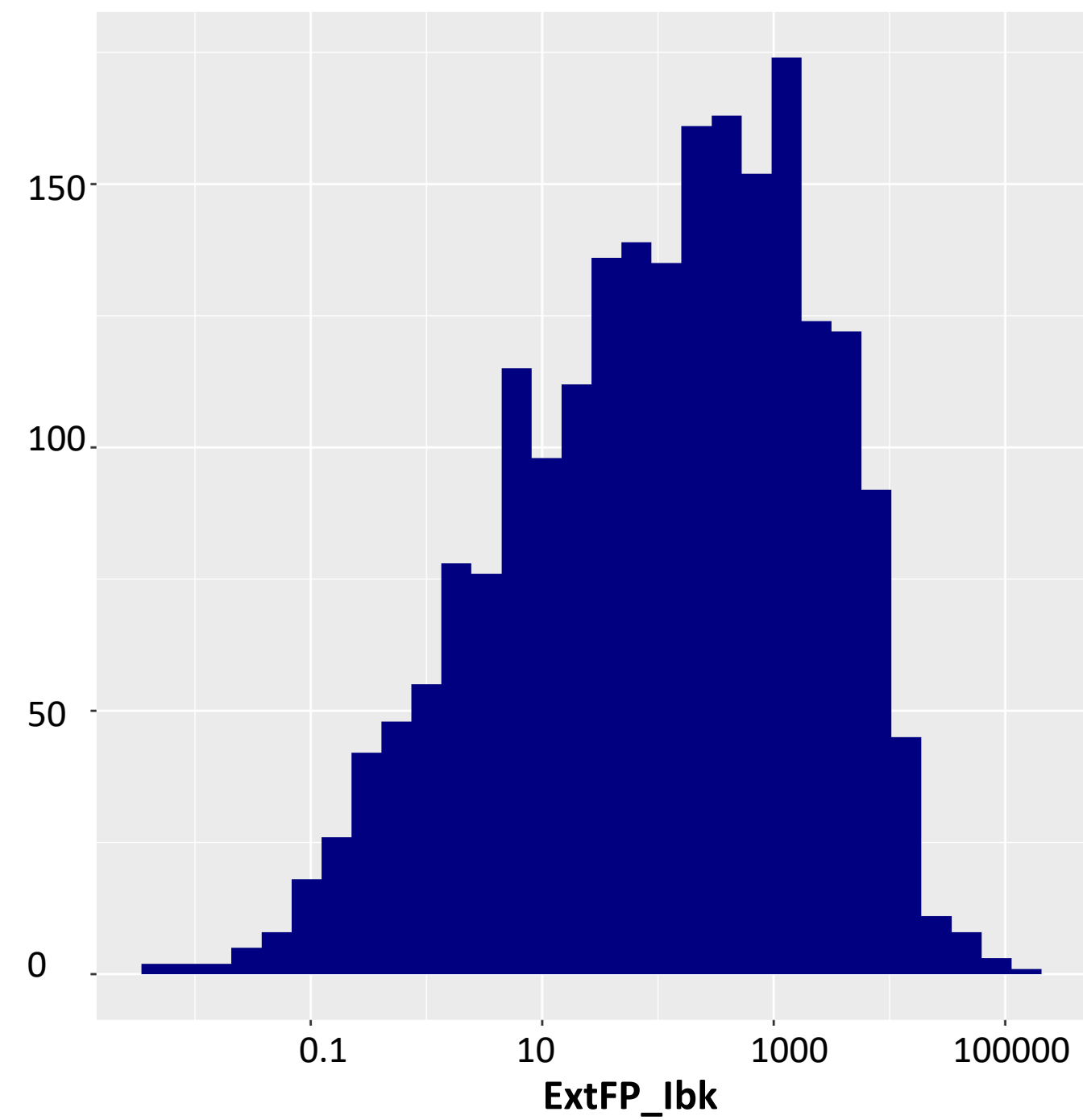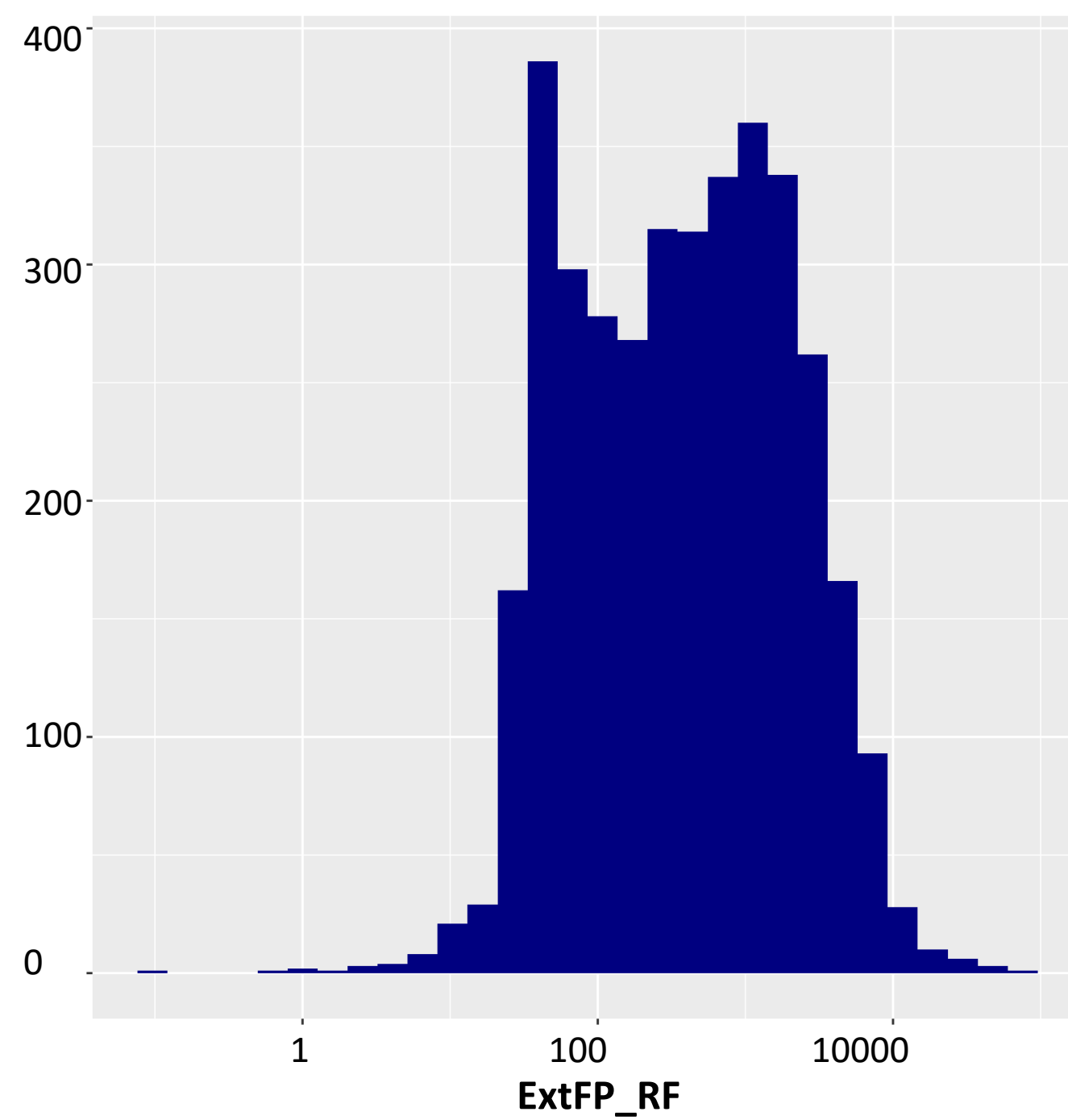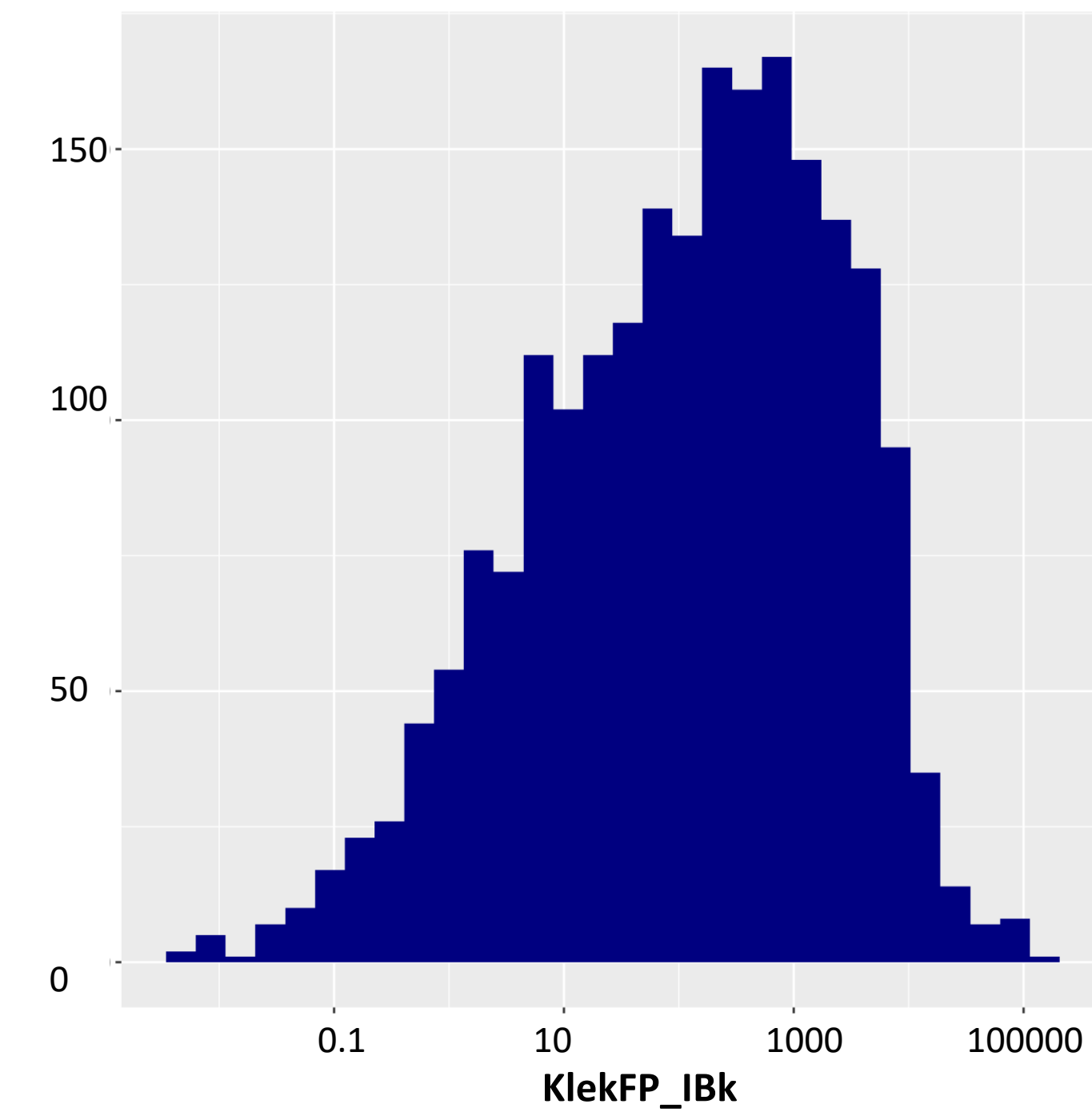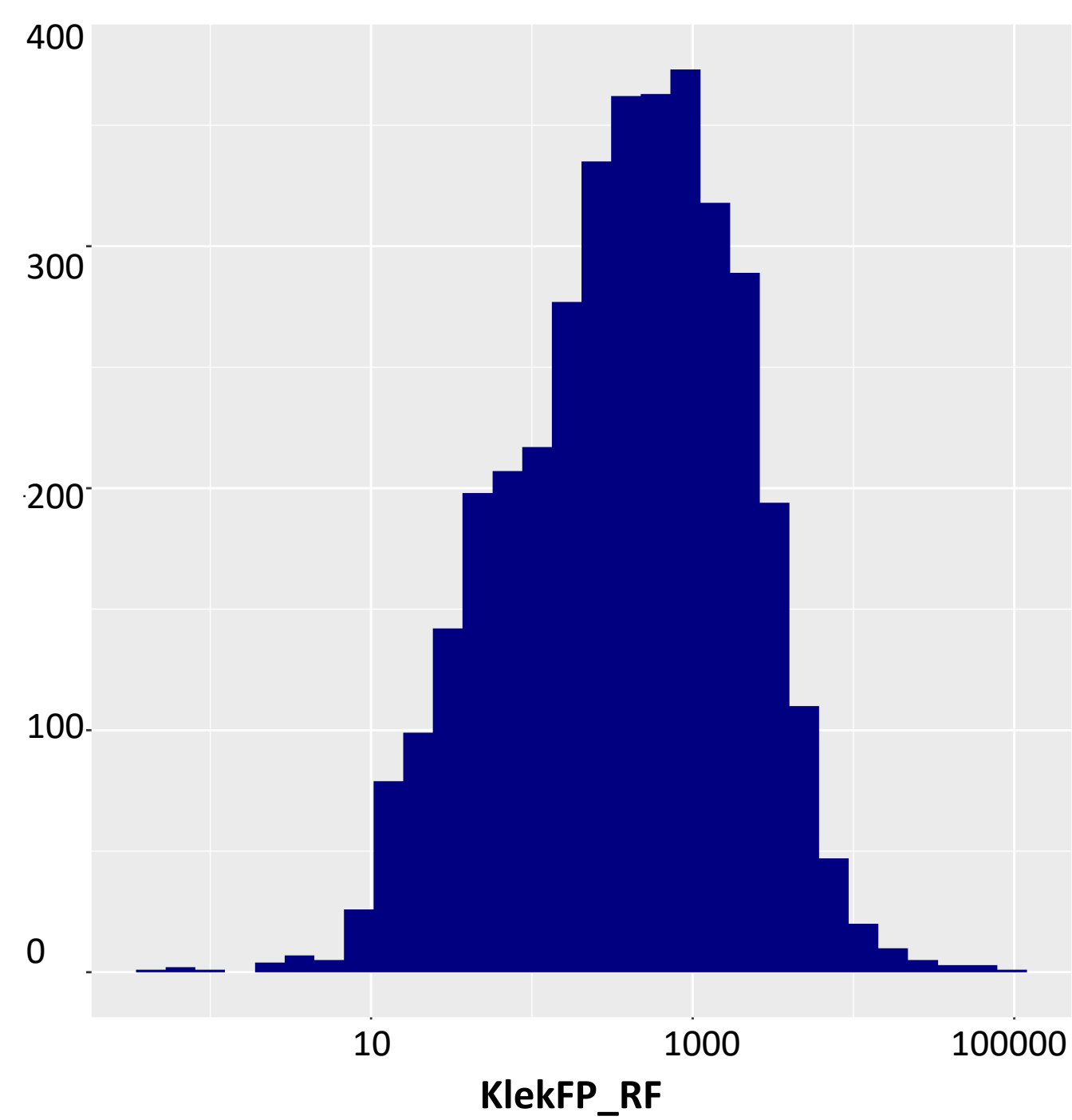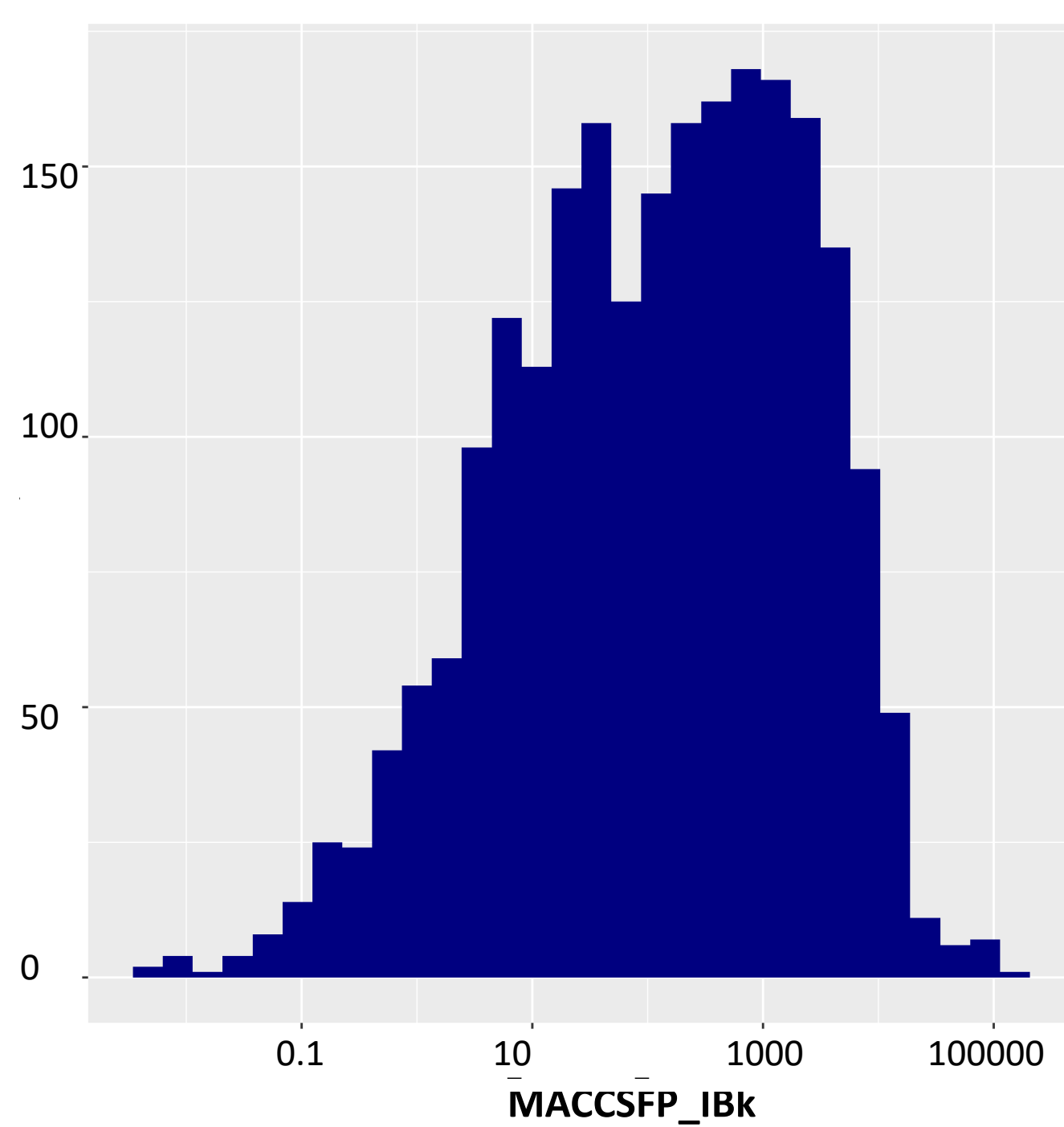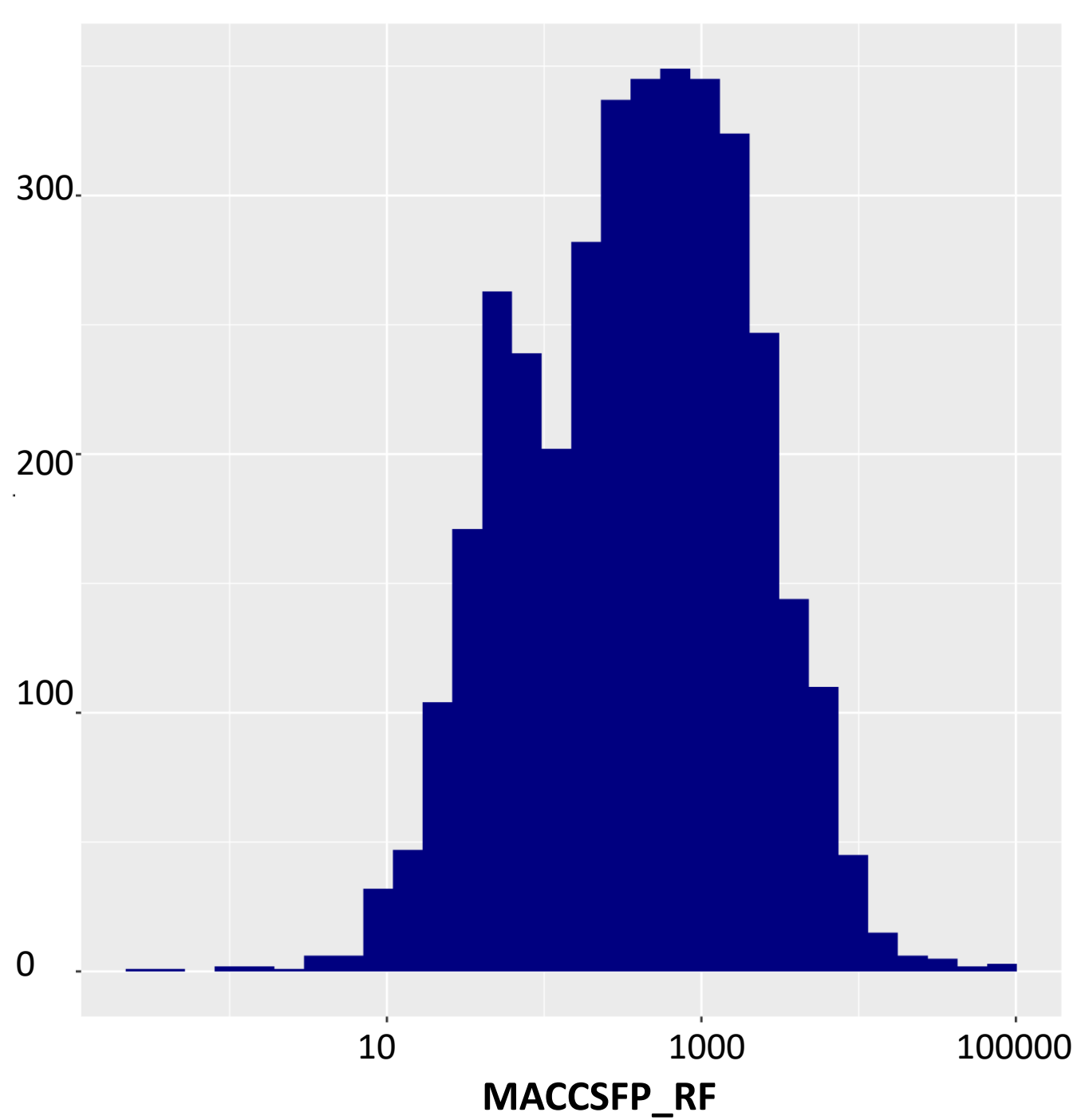

Supplement: Supplementary file 1 [file molecules-26-01607-s001.zip › histogram_prediction_error_mu.pdf]

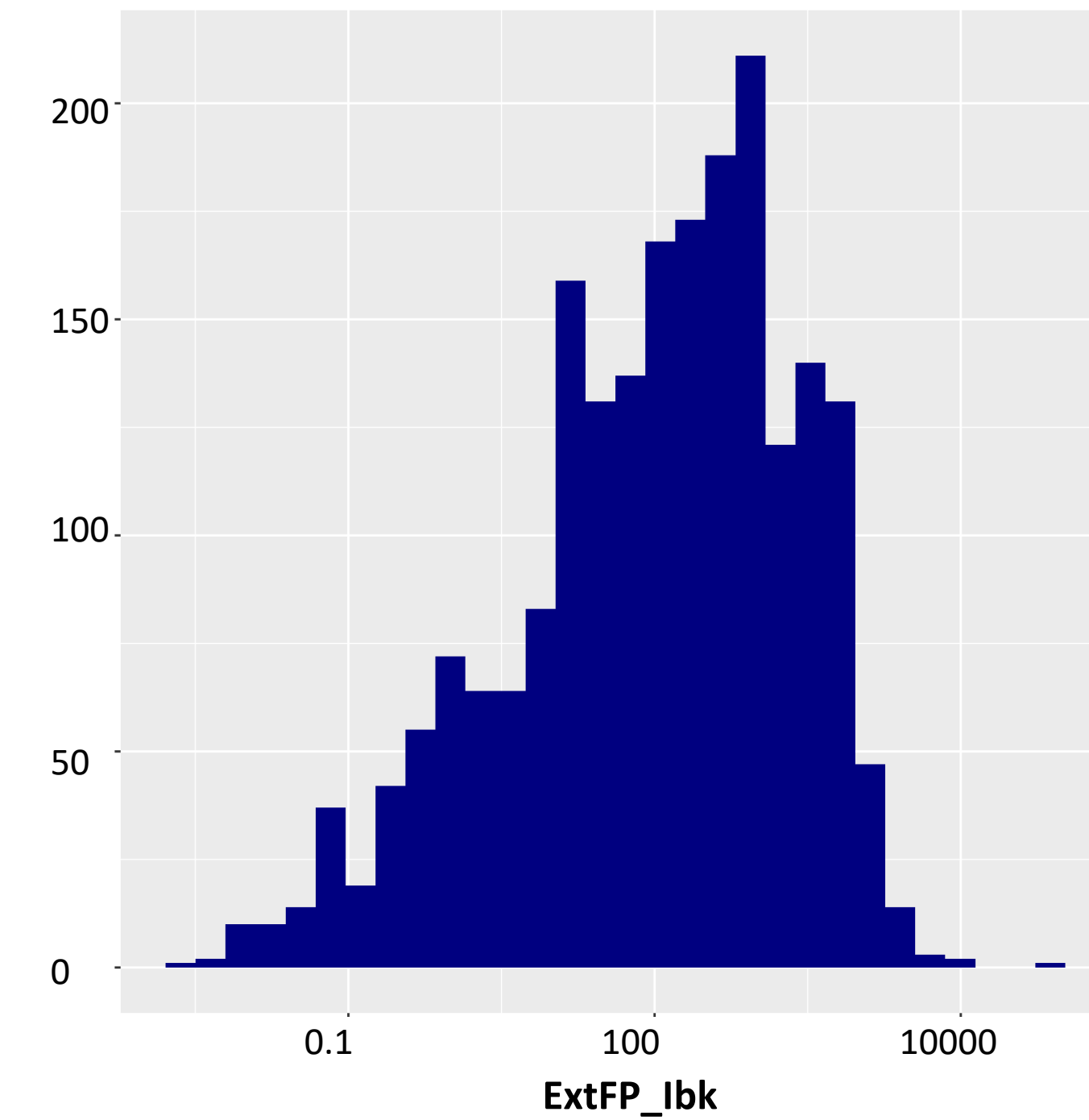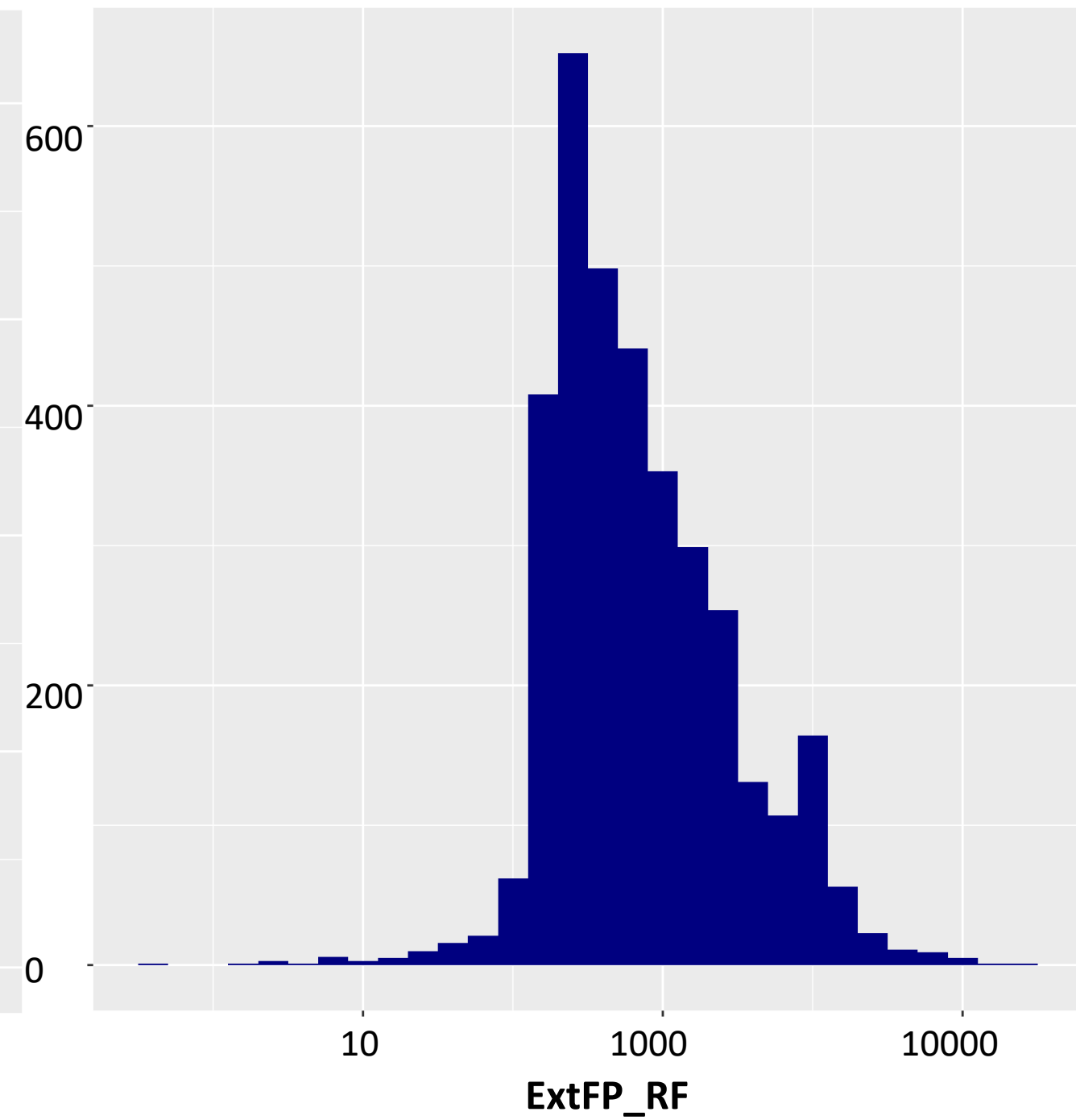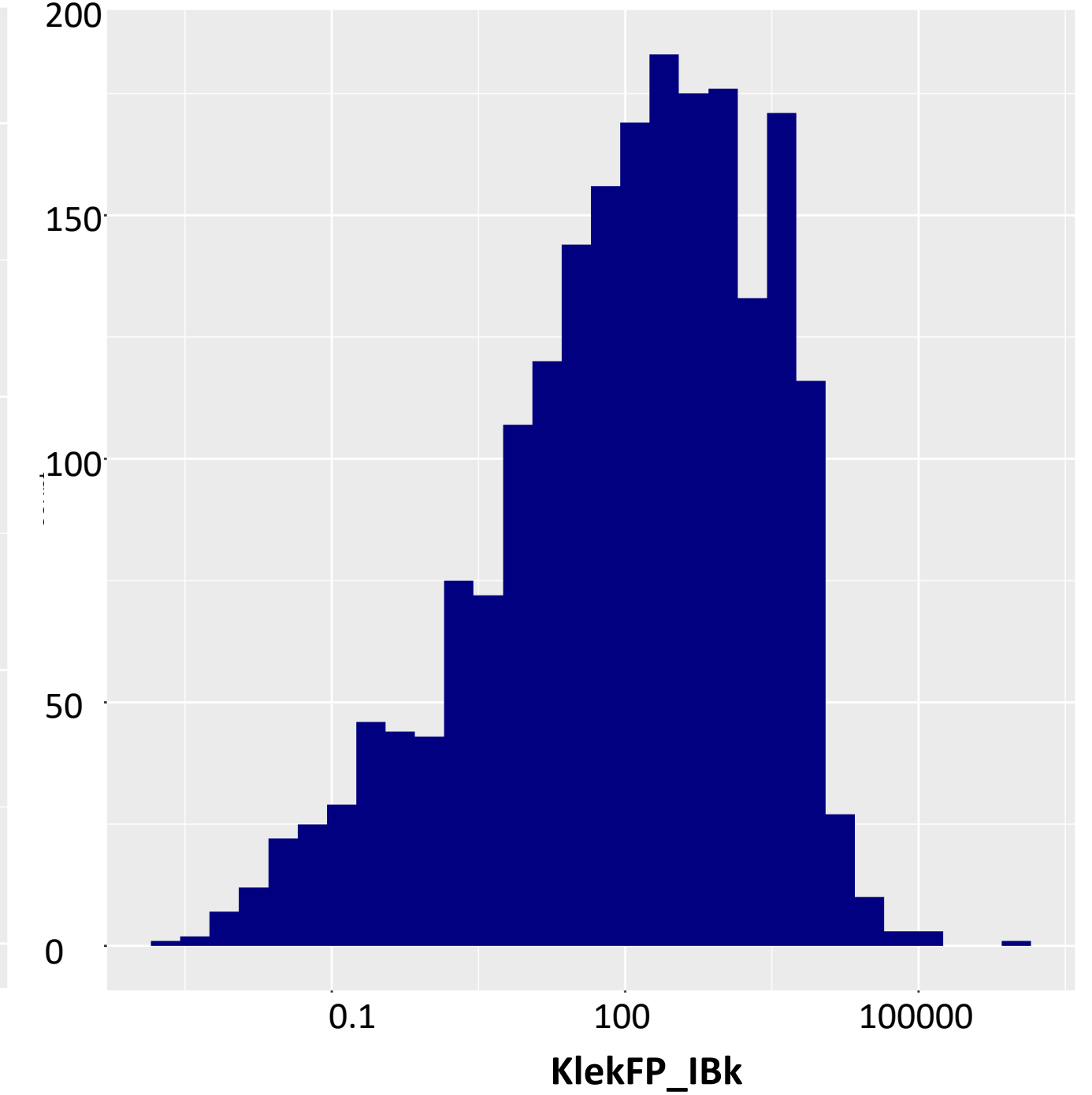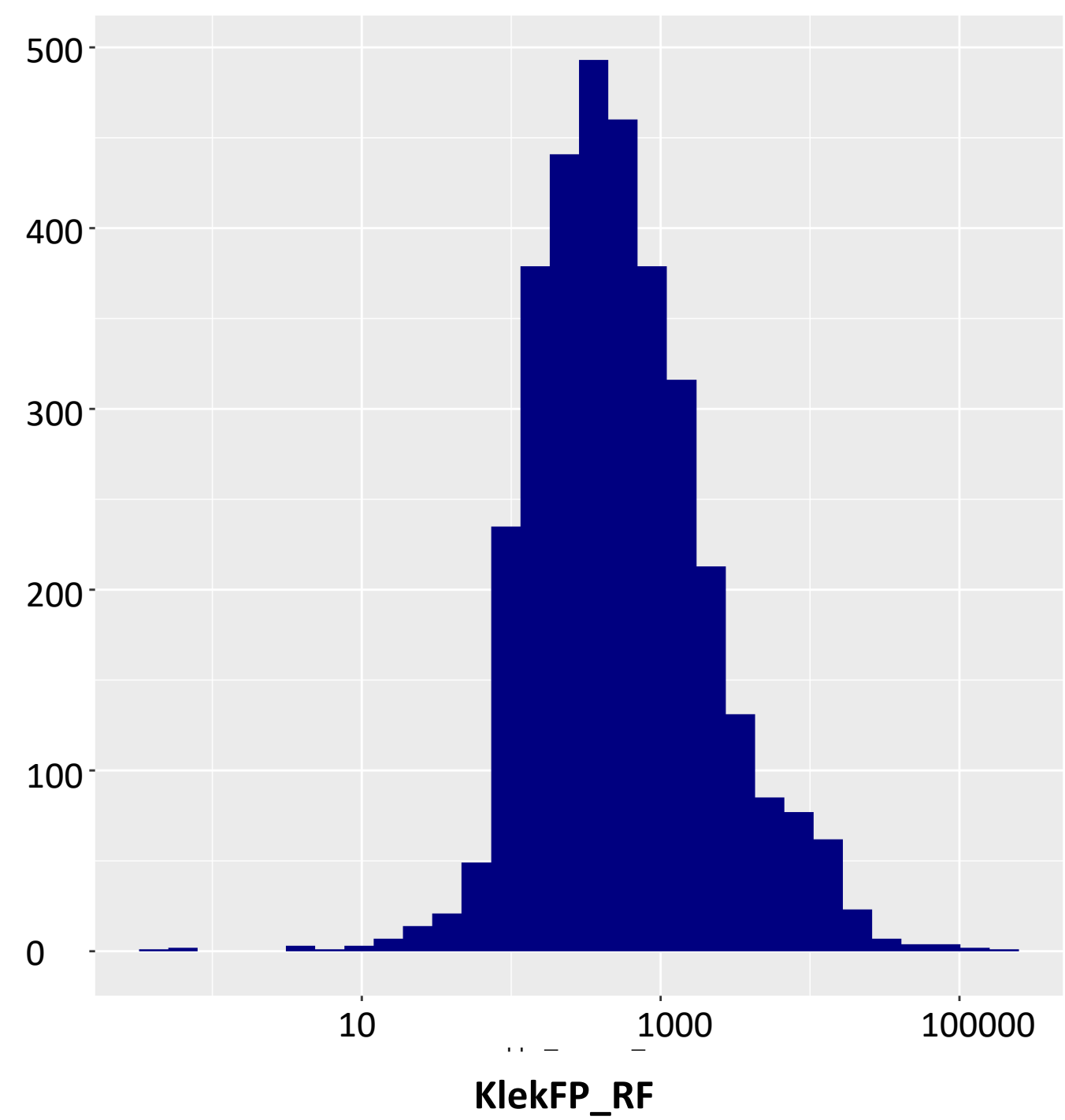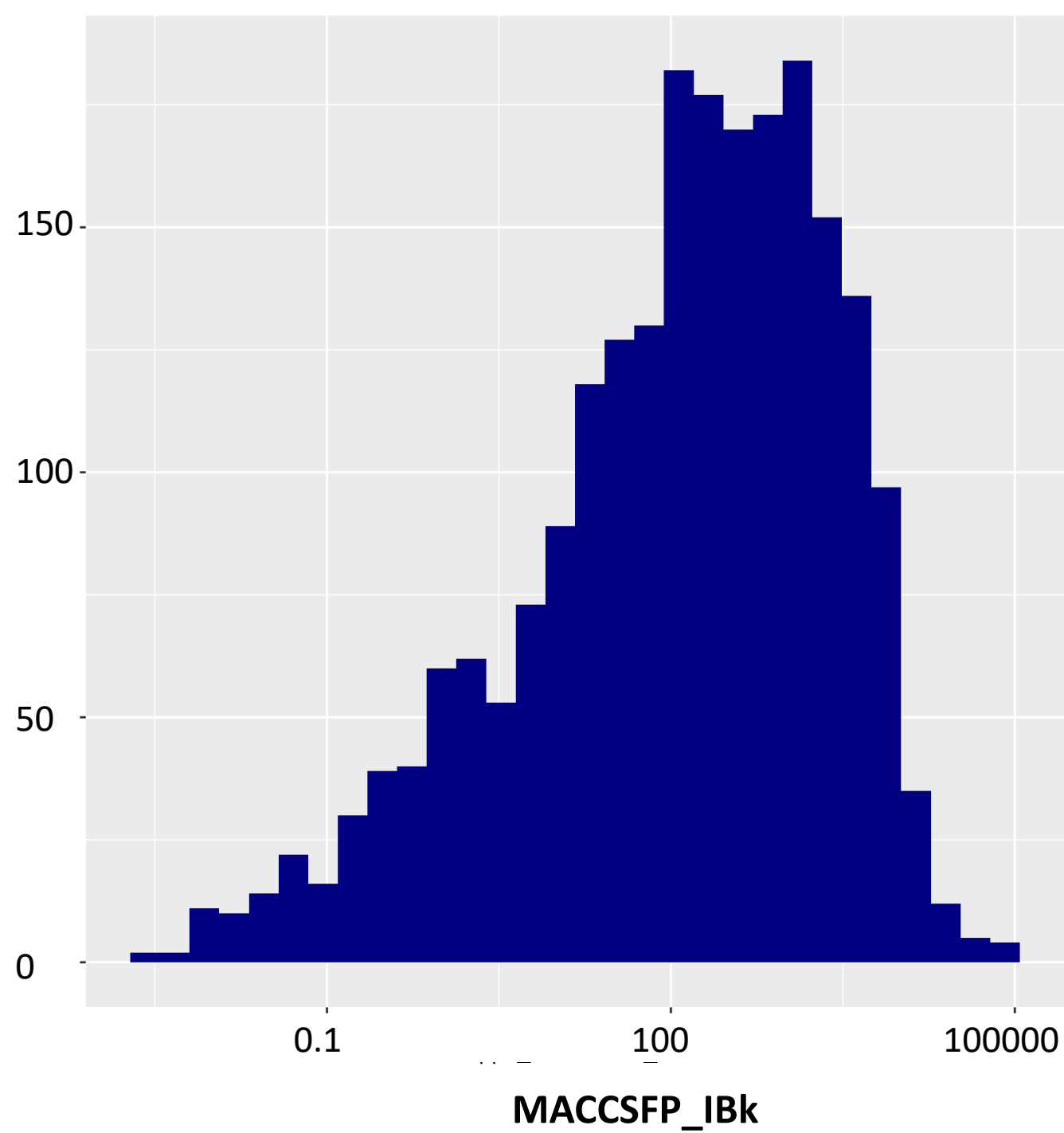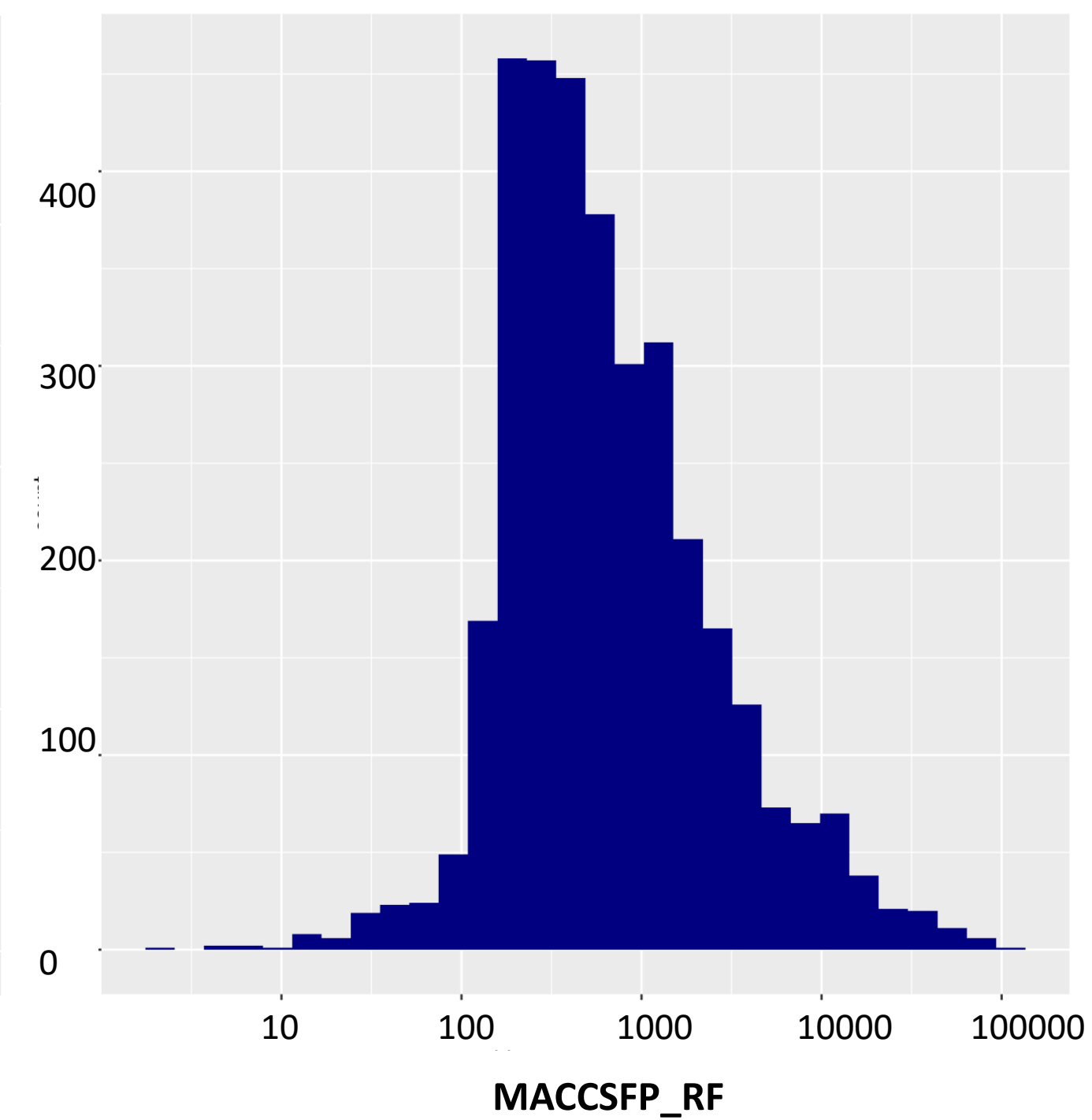

Supplement: Supplementary file 1 [file molecules-26-01607-s001.zip › histogram_prediction_error_kappa.pdf]
